# Identifying BAP1 Mutations in Clear-Cell Renal Cell Carcinoma by CT Radiomics: Preliminary Findings

**DOI:** 10.3389/fonc.2020.00279

**Published:** 2020-02-28

**Authors:** Zhan Feng, Lixia Zhang, Zhong Qi, Qijun Shen, Zhengyu Hu, Feng Chen

**Affiliations:** ^1^Department of Radiology, College of Medicine, The First Affiliated Hospital, Zhejiang University, Hangzhou, China; ^2^Department of Radiology, Hangzhou First People's Hospital, Hangzhou, China; ^3^Department of Radiology, Second People's Hospital of Yuhang District, Hangzhou, China

**Keywords:** CT, clear cell renal cell carcinoma, machine learning, BAP1 mutation, radiomics

## Abstract

To evaluate the potential application of computed tomography (CT) radiomics in the prediction of BRCA1-associated protein 1 (*BAP1*) mutation status in patients with clear-cell renal cell carcinoma (ccRCC). In this retrospective study, clinical and CT imaging data of 54 patients were retrieved from The Cancer Genome Atlas–Kidney Renal Clear Cell Carcinoma database. Among these, 45 patients had wild-type *BAP1* and nine patients had *BAP1* mutation. The texture features of tumor images were extracted using the Matlab-based IBEX package. To produce class-balanced data and improve the stability of prediction, we performed data augmentation for the *BAP1* mutation group during cross validation. A model to predict *BAP1* mutation status was constructed using Random Forest Classification algorithms, and was evaluated using leave-one-out-cross-validation. Random Forest model of predict *BAP1* mutation status had an accuracy of 0.83, sensitivity of 0.72, specificity of 0.87, precision of 0.65, AUC of 0.77, F-score of 0.68. CT radiomics is a potential and feasible method for predicting *BAP1* mutation status in patients with ccRCC.

## Introduction

Clear-cell renal cell carcinoma (ccRCC) is the most common kidney cancer in adults, and its pathogenesis is complicated. Fortunately, there are less significantly mutated genes in ccRCCs compared with other cancers (1); the top four most commonly mutated genes are von Hippel-Lindau (*VHL*) tumor suppressor gene, polybromo-1 (*PBRM1*), *BRCA1*-associated protein 1 (*BAP1*), and SET domain containing 2 (*SETD2*) (2, 3).

Even though *VHL* mutation occurs in as high as 52% of ccRCC cases, meta-analysis indicates that it has no prognostic or predictive value in patients with ccRCC (4). *BAP1* mutated in 10–15% of ccRCC (5), but it has recently garnered attention for several reasons. Brugarolasl et al. reported an association between *BAP1* mutation and pathology grading of ccRCC (6). Moreover, greater than 50% of patients with ccRCC with *BAP1* mutations exhibit coagulative tumor necrosis and have poor clinical outcomes (7). Other studies have demonstrated an association between *BAP1* mutation and mammalian target of rapamycin (mTOR) pathway activation (8, 9). Patients with *BAP1* mutation do not respond well to targeted therapy, and those with wild-type tumors appear to have longer progression-free survival than those with *BAP1* mutation tumors (10).

Tumor imaging phenotypes are closely associated with their gene expression patterns, protein, or other molecular changes (11). Radiogenomics analyze the relationship between imaging phenotype and gene expression patterns and provide insights into the genetic background and developmental status of the disease (5). Liu et al. utilized computed tomography (CT) imaging features to predict epidermal growth factor receptor (*EGFR*) mutations in patients with non-small cell lung cancer. Their results suggest that wild-type *EGFR* is associated with conditions such as emphysema and airway malformation, while *EGFR* mutations are associated with ground-glass opacity changes (12). In addition, the isocitrate dehydrogenase 1 (*IDH1*) gene mutation is considered a specific marker for glioma, and the radiomics method has been developed to reveal *IDH1* status for patients with glioma (13). Due to the fact that ccRCC with different genotypes may respond differently to targeted therapy, the extraction of imaging biomarkers that are capable of predicting *BAP1* mutation would be of great significance for ccRCC precision therapy (14, 15). In this study, we evaluated the potential application of the radiomics method in predicting *BAP1* mutation status in patients with ccRCC.

## Materials and Methods

### Study Subjects

The patients' genetic data were from The Cancer Genome Atlas–Kidney Renal Clear Cell Carcinoma (TCGA-KIRC) database (https://cancergenome.nih.gov/), while corresponding radiological data were from The Cancer Imaging Archive (TCIA) (16). There were 537 patients in the TCGA-KIRC database, among which only 267 had corresponding radiological data. The inclusion criteria were, respectively, enrolled in our study for assessment: (1) *BAP1* mutation status from TCGA were available (*BAP1* mutated or unmutated), (2) available CT images in TCIA (contrast enhancement). The CT images with obvious noises, post-operative CT images, and unusable CT images were excluded from the study. A total of nine patients with *BAP1* mutation and 45 patients with *BAP1* unmutation met these criteria and thus were included in this study. The demographic and clinical characteristics of the patients are presented in Table 1.

**Table 1:** Demographic and clinical characteristics of patients.

**Characteristic**	**Value**
Mean age (year)	62
Sex	
Female	25 (46.3%)
Male	29 (53.7%)
*BAP1* mutation	
Absent	45 (83.4%)
Present	9 (16.6%)
Nuclear grade	
Fuhrman I/II	18 (33.3%)
Fuhrman III/IV	36 (66.7%)
TNM	
I	20 (37.0%)
II	7 (13.0%)
III	17 (31.5%)
IV	10 (18.5%)

The data related to this study were all from the public database and were used solely for scientific research. Therefore, ethical approval was not required.

### Tumor Segmentation

Tumor segmentation was based on the IBEX software package developed using Matlab (17). The region of interest (ROI) was drawn along the inner border of tumor as much as possible. The ROI was first drawn on the maximum tumor dimension in the axial plane, and additional segmentations were then performed on the adjacent upper and lower slices with 3–4 slices skipping. At the beginning of the study, 10 cases were picked randomly and used for ROI analysis by two independent radiologists with more than 10 years of experience. Both radiologists were blinded to the *BAP1* mutation status. The inter-observer variability was evaluated using intra-class correlation coefficient (ICC). ROI extraction for the remaining images was analyzed by one of the radiologists. In this study, we only used images in the CT enhancement nephrographic phase because of better tumor visualization in this phase. It was relatively difficult to delineate the tumor ROI on the CT images that were unenhanced or in the corticomedullary phase.

### Texture Feature Extraction and Selection

Texture feature extraction and calculation were performed using IBEX from both the original and filtered images. The Laplacian of Gaussian (LoG) filter was used for image filtration, with sigma value of 2 and 8 mm denoting fine and coarse patterns, respectively. The extracted texture features included intensity histogram, intensity direct, gray-level co-occurrence matrix, neighbor intensity difference matrix, and gray-level run length matrix.

Each research center used different CT protocols, which affect the radiomic features (18, 19). Orlhac et al. (20) developed the ComBat compensation method, which realigns radiomic features distributions and facilitates multicenter radiomics studies. It is a data-driven method that pools data from different centers and protocols in a common space for compensation. It does not require resample of CT images and will not change the definition of radiomic features (20). Therefore, we used the ComBat function (https://github.com/Jfortin1/ComBatHarmonization) to harmonize multisite imaging data achieved in TCIA (21).

Texture features with low reproducibility were abandoned. Inter-observer variability was evaluated using the intraclass correlation coefficient (ICC). Features with ICC value > 0.85 were further analyzed.

Mann-Whitney U test was primarily used to reduce the number of irrelevant and redundant texture features, features with *p* < 0.05 were retained. The level of collinearity among the features was assessed using Spearman's correlation coefficient (*r*). (22). The features with the lowest collinearity (*r* < 0.8) with the other features remained in the study.

### Model Construction

In this study, the number of patients with *BAP1* mutation that met the inclusion criteria was very limited, which resulted in an imbalance between the mutation group and wild-type group. To address this problem, we performed data augmentation for the small-sized *BAP1* mutation group and performed downsampling for the large-sized *BAP1* wild-type group. This is an effective method to solve the common problem of imbalanced classes in machine-learning classification and has been well accepted in both academia and industry (23, 24). According to previous imbalanced data of radiomics study, each *BAP1* mutation case was segmented with more samples. After excluding the slices on the edge of the images, which could be affected by volume effects, each case generated 3–4 slices. By contrast, there were more cases with wild-type *BAP1*, so we randomly select some cases and generated two slices from each case. We initially had 54 ccRCC cases, which included 45 cases (90 segmentations) without *BAP1* mutations and nine cases (31 segmentations) with *BAP1* mutations.

Then, we used Synthetic Minority Over-sampling Technique (SMOTE) to analyze and simulate these data (24) and added these artificial samples to the new dataset. To avoid overfitting, the SMOTE is combined with cross validation (CV). Leave-one-out-cross-validation (LOOCV) is chosen for CV, this method is that we make use of all data points and hence it is low bias. When the sample size is small, LOCCV should be adopted to obtain a reliable accuracy estimate for a classification algorithm (25, 26). Specifically, in each iteration of LOOCV, after dividing a set of the original data as the validation set, SMOTE is used for the remaining training set. Therefore, for each LOOCV iteration, there were 180 labeled segmentations in training, which included 90 segmentations with wild-type *BAP1* and 90 segmentations with *BAP1* mutations.

**Figure 1:**
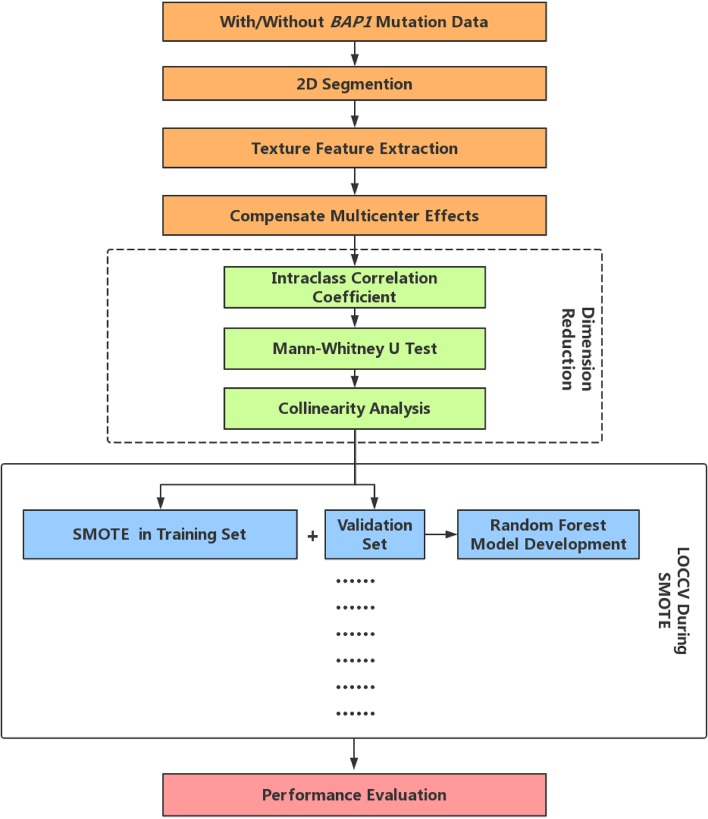
Radiomics analysis pipeline. LOOCV, Leave-one-out-cross-validation; SMOTE, Synthetic Minority Over-sampling Technique.

Random Forest (RF) is one of the most used machine-learning algorithms, because of its high performance and excellent generalization. In this study, the tree number of all RF classifiers was set to 500, Gini index was used to evaluate the importance of each feature. In LOOCV, RF first includes all the features after dimensionality reduction and then ranks the feature importance, then the first eight features in terms of importance are selected to reconstruct a new RF classifier. Finally, we performed a comprehensive evaluation of the constructed prediction model using commonly used cross-validated area under the curve (AUC) of receiver operating characteristic (27), accuracy, precision, recall, F-score (weighted harmonic mean of precision and recall), and Matthews correlation coefficient (MCC). The radiomics analysis pipeline is summarized in Figure 1.

## Results

**Figure 2:**
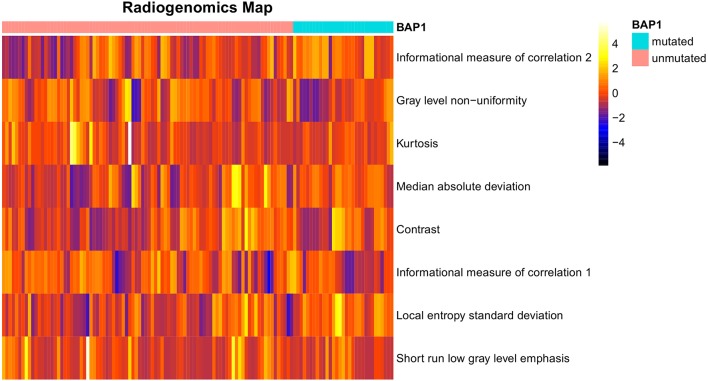
Radiogenomics map of selected features per mutation in the radiogenomics cohort. Each row represented a feature and each column represented a segmentation. The difference of each feature between *BAP1* mutated and unmutated can be observed.

After preliminary feature reduction, 58 features remain. Afterwards, the last feature that is involved in modeling for each LOCCV iteration is extracted and counted. The last features involved in RF modeling are summarized in Table 2 and Figure 2. Among the features used for model construction, most were extracted from LoG-filtered images, with a few extracted from the original images. Gray level run length matrix was the most selected radiomics feature classes. The most selected features for each model and their corresponding respective ICC values are provided in Table 2.

**Table 2:** Selected texture features for random forest classifiers.

**Feature Meaning**			**ICC**
**Image type**	**Feature class**	**Feature name**	
LoG filter (2 mm)	Intensity histogram	Median absolute deviation	0.93
No filter	Intensity histogram	Kurtosis	0.93
No filter	Gray level co-occurrence matrix	Informational measure of correlation 2	0.94
LoG filter (2 mm)	Gray level co-occurrence matrix	Informational measure of correlation 1	0.93
LoG filter (2 mm)	Gray level run length matrix	Gray level non-uniformity	0.97
LoG filter (2 mm)	Neighbor intensity difference	Contrast	0.97
No filter	Gray level run length matrix	Local entropy standard deviation	0.91
LoG filter (8 mm)	Gray level run length matrix	Short run low gray level emphasis	0.94

*LoG, Laplacian of Gaussian; ICC, intra-class correlation coefficient*.

The results showed that the RF-based predictive model had an accuracy of 0.83 [95% confidence intervals (CI): 0.76–0.88], sensitivity of 0.72 (95% CI: 0.65–0.79), specificity of 0.87 (95% CI: 0.82–0.93), precision of 0.65 (95% CI: 0.58–0.74), AUC of 0.77 (95% CI: 0.70–0.83), F-score of 0.68 (95% CI: 0.61–0.76), and MCC of 0.58 (95% CI: 0.50–0.66).

## Discussion

Our predictive model showed excellent performance in the dataset from TCGA. The results suggest that RF algorithm-based high-dimensional quantitative CT radiomics analysis might be a feasible and potential method for predicting *BAP1* mutation status in patients with ccRCC.

Radiomics has shown promise for the differentiation of pathological type, prediction of prognosis, and therapeutic response in ccRCC (28–30). However, radiogenomics in ccRCC has been limited. Karlo et al. investigated the association between CT features of ccRCC and mutations in *VHL, PBRM1, SETD2, KDM5C*, and *BAP1* genes (31). Their results showed that mutation of *BAP1* was significantly associated with evidence of renal vein invasion. Shinagare et al. (22) reported that *BAP1* mutation was associated with ill-defined margins and presence of calcification. However, these studies were based on qualitative CT image features. Shinagare et al. also noted that the definitions of some imaging features are hard to specify, consequently resulting in inconsistent conclusions among observers (22). Kocak et al. (32) conducted high-dimensional quantitative CT texture analysis in 45 patients with clear cell RCC (29 without *PBRM1* mutation and 16 with *PBRM1* mutation). The RF algorithm correctly classified 95.0% of the ccRCCs (32). These studies demonstrated that the characteristic gene signature of ccRCC accurately correlated with CT image phenotype.

Our research might be of more practical and clinical significance compared with previous studies. Among the top four most commonly mutated genes, *BAP1* is most critical for personalized precision therapy. ccRCC is typically considered insensitive to radiation therapy. However, *BAP1* loss might sensitize RCCs to radiation (9). In addition, ubiquitin ligase, which is closely associated with *BAP1* protein, is a good candidate therapeutic target. Currently, Histone deacetylase (HDAC) is that target ubiquitin ligase are being studied in a clinical trial for ccRCC treatment (14). Moreover, *BAP1* mutation sensitizes cells to poly (ADP-ribose) polymerase inhibitors and a clinical trial of an HDAC for the treatment of patients with refractory metastatic RCC is ongoing (33). High-throughput genome sequencing is gradually being used in the clinic; however, this technique is relatively expensive and requires special techniques. CT has become indispensable in clinical routine. Therefore, we postulated that developing a CT feature-based model to predict genotypes of ccRCC would be of great significance for precision medicine.

Regarding the previous works on imaging research of *BAP1* mutation based on TCGA and TCIA data, Ghost et al. found out that the prediction model based on nephrographic phase images performed the best with an area under curve (AUC) of 0.71 (34). However, they failed to make corresponding adjustments when the number of *BAP1* mutations is too few. Realizing that the processing for this batch of unbalanced data was the key to research, Kocak et al. (35) also adopted the strategy of oversampling to the *BAP1* mutation data. Regrettably, however, they merely conducted the unenhanced CT texture analysis (35). To design a machine learning model that can identify this rare genetic mutation in large amounts of ccRCC patients and make up for inadequacies in previous studies, nephrographic phase data is directly used in this research. In the oversampling section, the oversampling of *BAP1* mutation data is innovatively integrated into CV, which reduces the relevance between the data in the training set and the validation set to the fullest extent. And RF with an overbagging characteristic is chosen as the classifier, which also embeds partial feature selection in CV iteration. These methods are highly effective techniques for tackling an imbalanced dataset and may reduce the risk of overfitting.

This study had some limitations. First, it had some intrinsic downsides of a retrospective study design. Second, the sample size was limited, which may have caused overfitting during machine learning. We utilized some commonly used and effective techniques to address this problem. However, further validation of this prediction model using external datasets will be necessary. Third, we only analyzed two-dimensional texture features in this study. Three-dimensional texture features and morphological features were not analyzed. However, some studies have reported satisfactory results based on a single or few slices (30, 36, 37). Finally, it is not an uncommon problem in radiogenomic studies that the data are class-imbalanced and the number of variables is very large and greatly exceeds the number of samples (5).

In summary, our study demonstrated that CT radiomics has great potential in predicting *BAP1* mutation status in patients with ccRCC. However, further research using larger datasets will be needed before this technique can be used clinically. The preliminary results from this study provide a basis for further radiogenomic studies for RCC.

## Data Availability Statement

Publicly available datasets were analyzed in this study. The patients' genetic data were from The Cancer Genome Atlas-Kidney Renal Clear Cell Carcinoma (TCGA-KIRC) database (https://cancergenome.nih.gov/), while corresponding radiological data were from The Cancer Imaging Archive (TCIA) (https://www.cancerimagingarchive.net/).

## Ethics Statement

Ethical review and approval was not required for the study on human participants in accordance with the local legislation and institutional requirements. Written informed consent for participation was not required for this study in accordance with the national legislation and the institutional requirements.

## Author Contributions

ZF, ZH, QS, and FC: conception and design, writing, review, and revision of the manuscript. ZF, LZ, and ZQ: analysis and interpretation of data. FC: study supervision.

### Conflict of Interest

The authors declare that the research was conducted in the absence of any commercial or financial relationships that could be construed as a potential conflict of interest.
